# Supplementary material for: Background compensation revisited: Conserved phase response curves in frequency controlled homeostats with coherent feedback
Source: PLoS One. 2024 Sep 4;19(9):e0305804. doi: 10.1371/journal.pone.0305804 (PMC11373829; doi:10.1371/journal.pone.0305804)
Supplement: Attachment — Submitted filename: Response to Reviewers.pdf [file pone.0305804.s005.pdf]

# Changes made in the revised manuscript based on the comments by Reviewer #1

Changes made in the manuscript with respect to the comments by Reviewer #1 are indicated in red in the marked-up copy of the revised manuscript. All line references relate to the marked-up copy.

**Reviewer #1:** This is an interesting publication, and a strong contribution to the ongoing effort to understand biological control systems, in this case the responses of regulated biological oscillators. The work is carried out to a high technical standard and described clearly and completely.
**Response:** *Thank you for the encouraging comment!*

**Reviewer #1:** 1) There is a great deal of material covered, and the sheer number of figures and results could make it difficult for a reader to follow their way through the paper. I wonder if it would be possible to provide a preview/outline at the start of the Results section, to give the reader a sense of what is about to be covered, and where to seek out results of particular types. Something like a bulleted list of the types of perturbations and responses to be investigated, pointing out where each will be addressed? It could make navigating the manuscript less daunting, especially for a reader who might arrive looking for a specific type of controller/response's results. (Alternatively, perhaps a summary/overview at the end of the Results section, collecting and summarizing the results that have been presented.)
**Response:** *I completely agree. In the original submission I had a section 'Outline' at the beginning of 'Results and Discussion' to give the reader an indication of the paper's content. I have now extended it. I renamed it 'Structure of the paper' and placed it just prior to 'Results and Discussion' (starting line 120). There I have made a more detailed description of the structure of the paper with a bulleted list of the main stages for each of the two controllers. I have also changed the section headings accordingly and rearranged sections such that the reader is able to associate parts of the paper with the bulleted list.*

**Reviewer #1:** 2) The main body of the text uses the first person pronoun, which makes perfect sense for a single-author paper. But the Abstract uses "we"; should that also be "I"? This is an actual question rather than a recommendation: I'm really not sure which is more correct, in an Abstract. It may be a question for the editors rather than the author.
**Response:** *I agree, the "we" form in the abstract may give a wrong association. I removed "We found that" and started the sentence with "Background compensation occurs...". I feel this solves it.*

# Changes made in the revised manuscript based on the comments by Reviewer #2

Changes made in the manuscript with respect to the comments by Reviewer #2 are indicated in blue in the marked-up copy of the revised manuscript. All line references relate to the marked-up copy.

**Reviewer #2:** An interesting manuscript. The results are valuable and are clearly articulated. I would recommend expanding the motivation and biological context to strengthen the case that this work is directly related to behaviours of regulated biomolecular oscillators. I also recommend streamlining the presentation by removing some of the figures (or moving them to the supplement). I've made specific recommendations below.
**Response:** *Thank you for your positive and constructive comments! Concerning motivation and the biological context/relevance of the results I have added a section 'Motivation and aim of this work' directly after the*

*Introduction (starting line 23). My motivation is related to the astonishing capability of seabirds and other animals living in large noisy colonies to recognize specific calls despite the ambient noise, which I feel may be related to the newly found property of background perturbation. The aim of the paper, on the other hand, is more technical, i.e. to understand the coherent feedback's response when inflow and outflow perturbations are applied at different backgrounds showing that frequency resetting and phase shifts are background compensated for both inflows and outflows.*
*Concerning the biological relevance I added a passage about this at the end of 'Results and discussion' (starting line 400). There is presently no evidence for this background compensation in the biological literature, as far as I can see. For completeness, I have mention PRC examples which show similar/analogous behaviors, but involve temperature as background combined with dark/light pulses.*

**Reviewer #2:** P3: It would be worthwhile to provide some review/context for motif indices from ref 31.?
**Response:** *Starting line 71 I have now added a section 'Negative feedback structures used in this study', where I briefly mention the eight 2-component negative feedback motifs M1-M8 and that they fall into two classes termed inflow and outflow controllers. Then I mention the two motifs used in the study, which will be covered in detail later in the manuscript.*

**Reviewer #2:** The justification for approx. 1 in eqn 9 is not clear (i.e. why k7 $\ll$ Ess is guaranteed.)
**Response:** *Starting line 158 I have now included a brief description that enzymes which show zero-order kinetics bind strongly to their substrates and have low $K_M$ values. Then I include the statement that in the calculations zero-order conditions are generally assured when $K_M$, i.e. $k_7$ here, is assumed to be in the order between $10^{-4}$ and $10^{-6}$ au. I have also included a reference to Fig S9 in Ref 20, which shows that a controller's homeostatic accuracy is diminished when the $K_M$ is gradually increased.*

**Reviewer #2:** P7-8 figs 7-8-9. I had trouble following the argument that 'While Fig 9 provides insights into why the resetting of k1 steps is markedly 195 different from those of k2 steps.' The responses in fig 9 are not qualitatively different. Is the comparison of size of response in 6D and 7D the most relevant feature here? Some additional guidance in interpreting these figures (which are dense) would be useful.
**Response:** *I see that this part of the manuscript is terse. From line 233 (starting with "In case of the $k_1$ step......") to line 240 I have now included a more detailed description of the differences between $k_1$ and $k_2$ perturbations which lead to the differences observed in Figs 6a and 6b.*

**Reviewer #2:** P8: Similarly: "I tested whether background 196 compensation is operative for both k1 and k2 step perturbations. This is shown in Figs 10 and 11." More guidance is needed to interpret these results.
**Response:** *I have now made the description more explicit (see lines 243-247) stating that both backgrounds show identical frequency resettings, since orange and blue dots lie precisely at the same locations.*

**Reviewer #2:** P9: Fig 12 is perhaps not needed: PRC construction has already been illustrated. Likewise Figures 14, 24 and 25
**Response:** *I thought about this, but decided to keep the figures. Partly to show that transients in the resetting can be quite different as seen in Figs 12 and 14, but also because I refer to these figure legends from the captions of Figs 13 and 15. Since PLOS ONE has no space restrictions I feel it is simplest to keep the figures as they are.*

**Reviewer #2:** P10: "Surprisingly, this constant phase shift zone resembles that of a dead zone," Can anything more be said about this? Seems a remarkable result.

**Response:** *Concerning the results in Fig 15 and Fig 25 I searched a collection of PRCs compiled by Carl Johnson (the PRC Atlas), and found only a few PRCs which appear similar to Fig 15 and Fig 25. I added a paragraph (starting at line 400) at the end of 'Results and discussion' where these PRCs are mentioned with references, but there is no evidence for the type of background compensation described in the paper.*
*There have been a few experiments where temperature was used as a background, but PRCs were done with light- or dark-pulse perturbations. There, background compensation was observed for two temperature values, but since both temperature and light have been involved, these type of experiments were different from the situation I am considering here, where background and perturbation acts on the same variable/reaction channel. Starting line 406 I have, for the sake of completeness, described briefly these types of PRCs with references, but pointed out the difference with respect to my findings.*

**Reviewer #2:** P12: space needed :' are changed,respectively."
**Response:** *Thank you. This has been corrected.*

## Additional changes made in the revised manuscript

Additional changes, rewordings, etc. are outlined in violet.

- Lines 169-171: As part of the description of M2, I have added that M2 is an inflow controller, i.e. the compensatory flux $j_3$ is an inflow to $A$, thereby compensating outflows from the controlled variable $A$.

- Starting line 429: With respect to the outlook, I have added reference 67, which describes brain feedbacks with the ability to actively adjust and improve auditory signal processing. To what extent such processes may contribute to background compensation (for example of ambient noise) needs to be seen.

- Due to the restructuring of the manuscript in response to the comment of Reviewer #1 the following figure numbering has changed:
Fig 18 becomes Fig 21 in revised version
Fig 21 becomes Fig 18 in revised version
Fig 19 becomes Fig 22 in revised version
Fig 22 becomes Fig 19 in revised version
Fig 20 becomes Fig 23 in revised version
Fig 23 becomes Fig 20 in revised version

- In panels b of Figs 7 and 8 the average $<E>$ calculated by Eqs 2 and 3 (outlined in blue) was by a mistake labeled as $<E>_i$, which is now corrected to $<E>$. For the sake of completeness I added in the legend of Fig 8 the sentence:
*Averages $<A>$ and $<E>$ (green line) are calculated by Eq 1 while $<E>$ values outlined in blue are calculated by Eqs 2 and 3 as in Fig 7.*
