# Supplementary material for: Background compensation revisited: Conserved phase response curves in frequency controlled homeostats with coherent feedback
Source: PLoS One. 2024 Sep 4;19(9):e0305804. doi: 10.1371/journal.pone.0305804 (PMC11373829; doi:10.1371/journal.pone.0305804)
Supplement: S1 Program — A zip-file with Python and Matlab scripts showing results from Figs 3, 5, 7, 8, 12, 14, 17, 19, and 24. (ZIP) [file pone.0305804.s001.zip › S1 Programs/Fig14 (A-oscillations, matlab).zip!/Fig14 (A-oscillations, matlab)/Fig14a/k1 1--> 128 step/Figure14a_perturbed.pdf]

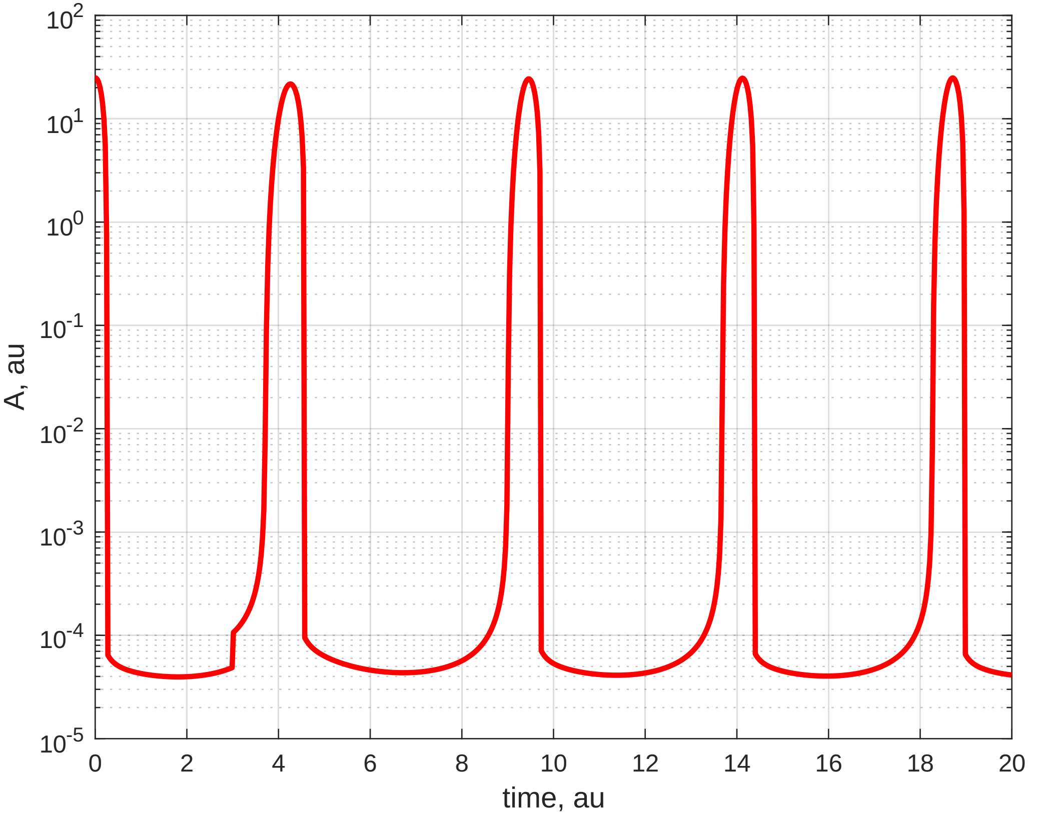

10^2
10^1
10^0
10^-1
10^-2
10^-3
10^-4
10^-5
A, au
0
2
4
6
8
10
12
14
16
18
20
time, au
