# Supplementary material for: Background compensation revisited: Conserved phase response curves in frequency controlled homeostats with coherent feedback
Source: PLoS One. 2024 Sep 4;19(9):e0305804. doi: 10.1371/journal.pone.0305804 (PMC11373829; doi:10.1371/journal.pone.0305804)
Supplement: S1 Program — A zip-file with Python and Matlab scripts showing results from Figs 3, 5, 7, 8, 12, 14, 17, 19, and 24. (ZIP) [file pone.0305804.s001.zip › S1 Programs/Fig7 (python).zip!/Fig7 (python)/plots/I12-time-plot_fig7.pdf]

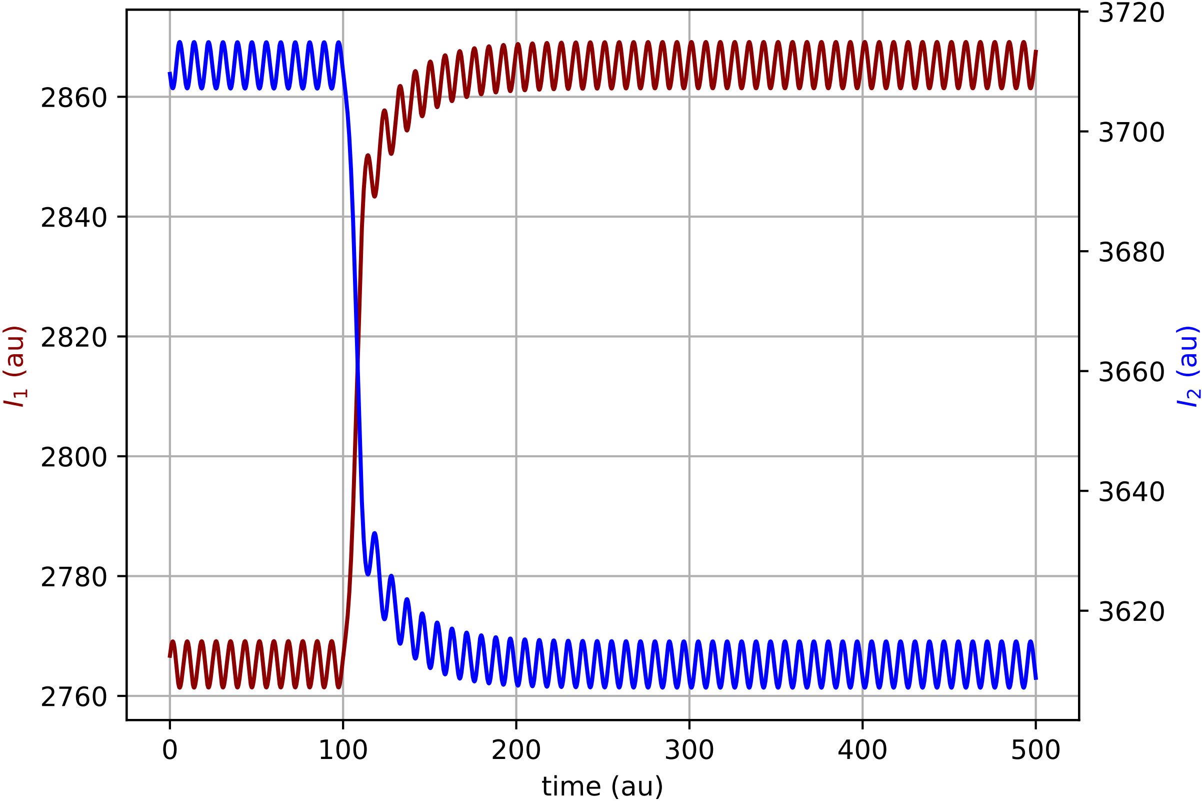

2860
2840
2820
2800
2780
2760
3720
3700
3680
3660
3640
3620
0
100
200
300
400
500
time (au)
I_1 (au)
I_2 (au)
