# Supplementary material for: Background compensation revisited: Conserved phase response curves in frequency controlled homeostats with coherent feedback
Source: PLoS One. 2024 Sep 4;19(9):e0305804. doi: 10.1371/journal.pone.0305804 (PMC11373829; doi:10.1371/journal.pone.0305804)
Supplement: S1 Program — A zip-file with Python and Matlab scripts showing results from Figs 3, 5, 7, 8, 12, 14, 17, 19, and 24. (ZIP) [file pone.0305804.s001.zip › S1 Programs/Fig12 (A oscillations, python).zip!/Fig12 (A oscillations, python)/panel a/plots/A-time-plot_fig12a_perturbed.pdf]

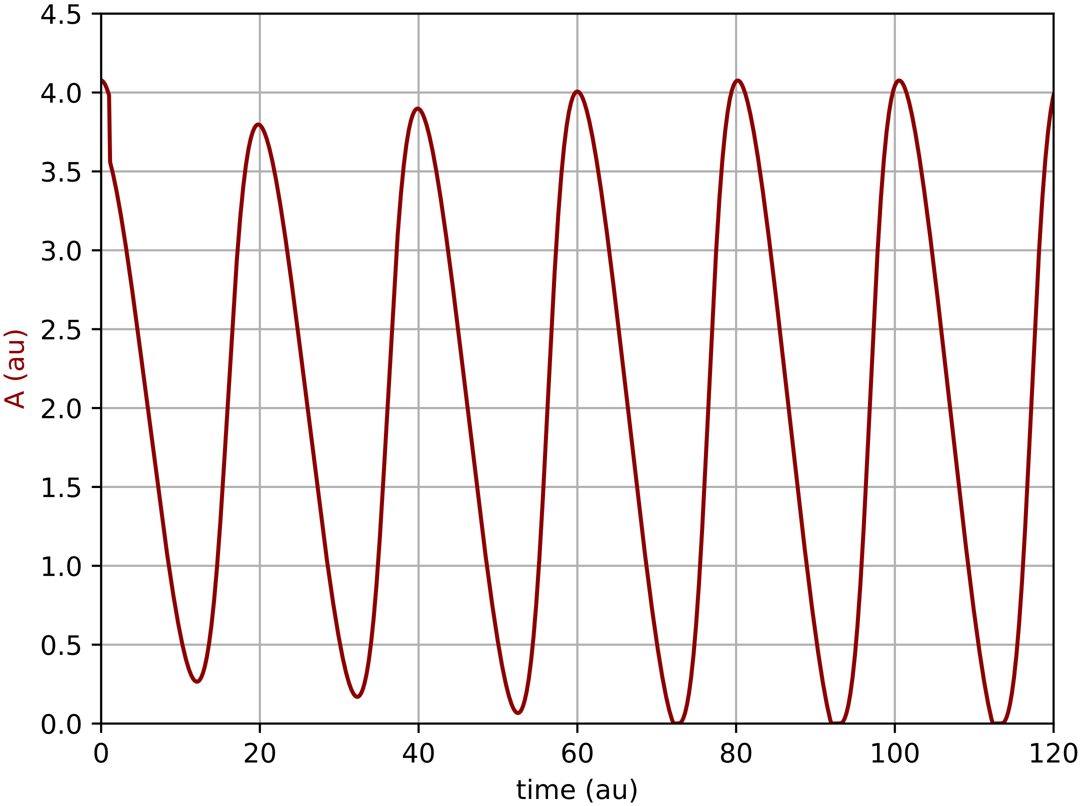

4.5
4.0
3.5
3.0
2.5
2.0
1.5
1.0
0.5
0.0
A (au)
0
20
40
60
80
100
120
time (au)
