# Supplementary material for: Background compensation revisited: Conserved phase response curves in frequency controlled homeostats with coherent feedback
Source: PLoS One. 2024 Sep 4;19(9):e0305804. doi: 10.1371/journal.pone.0305804 (PMC11373829; doi:10.1371/journal.pone.0305804)
Supplement: S1 Program — A zip-file with Python and Matlab scripts showing results from Figs 3, 5, 7, 8, 12, 14, 17, 19, and 24. (ZIP) [file pone.0305804.s001.zip › S1 Programs/Fig24 (A oscillations, python).zip!/Fig24 (A oscillations, python)/panel b/panel b (k1b=90, k2b=5.0)/plots/A-time-plot_fig24b.pdf]

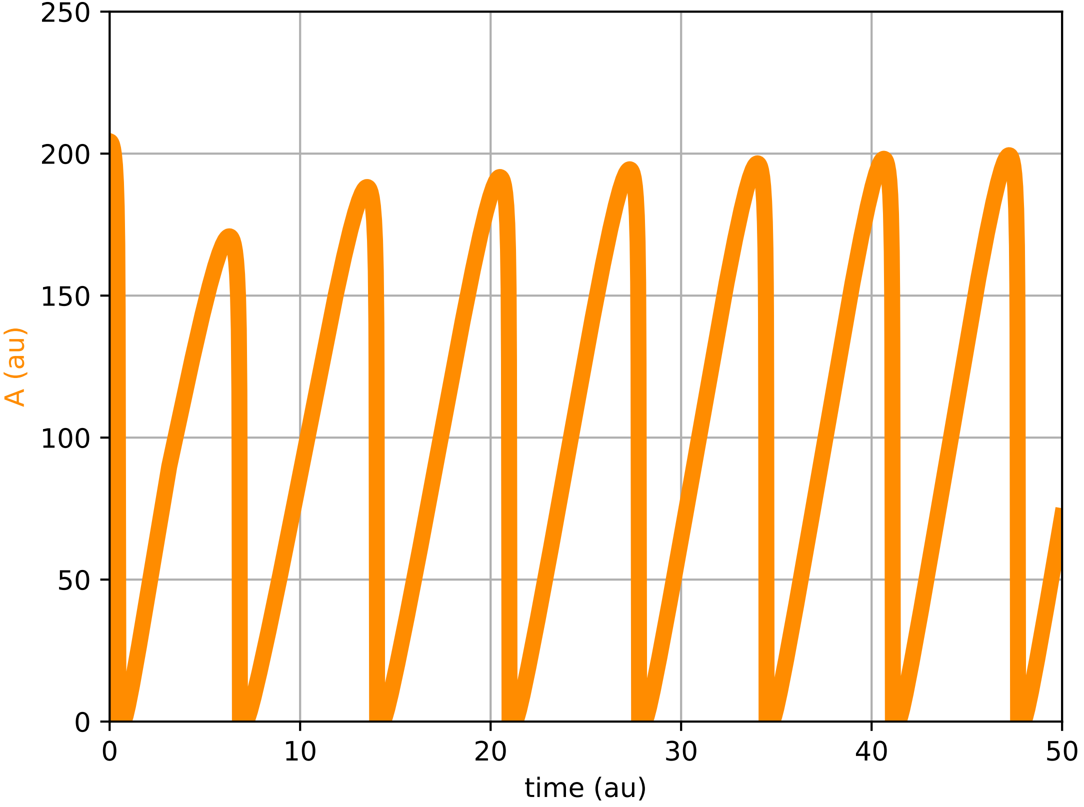

250
200
150
100
50
0
A (au)
0
10
20
30
40
50
time (au)
